# Angiotensin converting enzyme inhibitors and angiotensin receptor blockers impact on the gut microbiome: a systematic review

**DOI:** 10.3389/fendo.2025.1685424

**Published:** 2025-10-23

**Authors:** Elisabeth M. Wang, Abdulwhab Shremo Msdi, Vy N. Quach, Selena Q. Nguyen, Emily Quach, Jinhee Jo, Taryn A. Eubank, Kevin W. Garey, Natalie Rosario

**Affiliations:** College of Pharmacy, University of Houston, Houston, TX, United States

**Keywords:** hypertension, ACEi (angiotensin-converting enzyme inhibitor), ARB (angiotensin II receptor blocker), microbiome, dysbiosis

## Abstract

**Background:**

Inhibition of the renin–angiotensin system (RAS) may influence gut microbial composition and blood pressure, yet current evidence remains limited. This review examines how angiotensin-converting enzyme inhibitors (ACEis) and angiotensin receptor blockers (ARBs) modify gut microbiome composition, function, and blood pressure regulation.

**Methods:**

We conducted a systematic search of MEDLINE and EMBASE from inception to September 2025 using terms including “human,” “rat,” “angiotensin converting enzyme inhibitor,” “angiotensin receptor blocker,” and “gut microbiome.” Eligible studies were required to report changes in microbiome diversity, bacterial composition, or short-chain fatty acids (SCFAs) associated with ACEi/ARB treatment across animal or human models. Data extraction and risk of bias assessments were performed independently by multiple reviewers.

**Results:**

After deduplication, 642 retrieved articles were filtered and nine met inclusion criteria (eight in rodent models, one human study). ACEi/ARB administration in animals was associated with increased microbial diversity, restoration of intestinal oxygen balance, and enrichment of SCFA-producing anaerobic genera such as *Bifidobacterium*, *Bacteroides*, *Blautia*, and *Akkermansia*. In the human study, ACEi/ARB use did not significantly alter microbial diversity, but decreased populations of facultative aerobic pathogens including *Staphylococcus* and *Enterobacterales*. Functionally, prolonged RAS inhibition elevated levels of acetate, propionate, and butyrate, and enhanced gut barrier integrity while attenuating inflammatory signaling. The human study was found to have a moderate risk of bias.

**Conclusions:**

ACEi and ARB therapies appear to reshape gut microbiome structure and metabolic function, promoting SCFA-producer expansion, improved gut barrier integrity, and modulation of microbial taxa linked to inflammation and hypertension. However, human data is limited, and further transitional research is needed to confirm these findings.

## Graphical Abstract

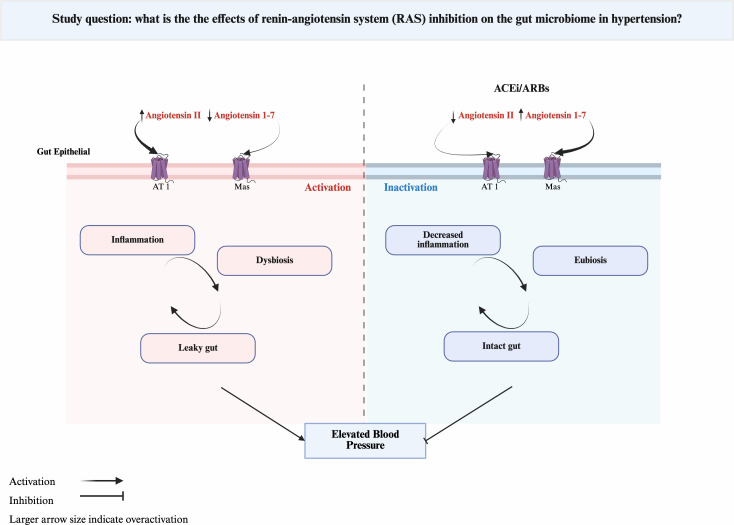

## Introduction

Hypertension (HTN), a leading cause of cardiovascular morbidity and mortality including stroke and myocardial infarction, affects approximately 122 million adults in the United States (US) (1–3). Less than 44% of American adults with hypertension have controlled blood pressure (BP), underscoring the urgent need for novel therapeutic strategies (1). The gut microbiota can impact HTN development and progression with altered gut microbial composition and function associated with elevated BP (4–7). Proposed mechanisms for this relationship include modulation of the renin-angiotensin system (RAS), vascular resistance, and immune responses (8–11).

The RAS, a key regulator of BP, comprises two opposing pathways: the ACE1-Ang II axis, which promotes vasoconstriction, inflammation, and fibrosis, and the ACE2-Ang 1–7 axis, producing counteracting effects (12). Traditionally, RAS was considered to function primarily as a systemic regulator of cardiovascular homeostasis via circulating Angiotensin II (Ang II). However, nearly all components of RAS have been identified in various local tissues, including the gastrointestinal tract (GIT) (13). Locally, Ang II has been shown to drive intestinal inflammation, increase gut permeability, and contribute to dysbiosis (14). Conversely, Ang 1–7 has demonstrated protective effects by restoring gut barrier integrity (15). Certain gut microbes such as *Bifidobacterium bifidum*, have direct angiotensin-converting enzyme (ACE) inhibitory activity, suggesting a direct influence on RAS activity (9). These observations suggest a complex bidirectional interplay between RAS signaling, gut health, and BP regulation.

RAS inhibitors, including angiotensin-converting enzyme inhibitors (ACEis) and angiotensin receptor blockers (ARBs), are cornerstone therapies for HTN management (16). Besides their hypotensive effect, growing evidence suggests that ACEis and ARBs may also influence gut health (17, 18). For example, candesartan has been shown to restore gut barrier integrity, enhance short chain fatty acid (SCFA) production, and increase microbial diversity in spontaneously hypertensive rats (SHR) (17). Despite these promising findings, evidence is limited and heterogenous. Importantly, the mechanisms by which RAS inhibition influences the gut microbiome are not well understood, highlighting a significant gap in literature. Exploring these mechanisms could expand our understanding of the actions of RAS inhibitors and reveal possibilities for innovative interventions using the gut microbiome to improve BP regulation. The aim of this systematic review was to assess the effects of ACEIs and ARBs on the composition, functionality, and pathology of the gut microbiome in hypertensive animal models and human studies.

## Methods

The Preferred Reporting Items for Systematic Review and Meta-Analyses (PRISMA) guidelines were used to conduct this systematic review (19). Inclusion criteria was the following: (1) hypertensive human or animal models; (2) ≥ 20 years old if human population; (3) use of ACEi/ARB as antihypertensive monotherapy; (4) evaluated at least 1 pre-specified outcome and (5) published in English. Systematic reviews, meta-analyses, *post-hoc* analyses, and methodology articles were excluded. Outcomes of interest included changes in gut composition and function, evaluated by alpha and/or beta diversity, bacterial taxa, and SCFAs, along with gastrointestinal health and inflammatory changes related to ACEi or ARB treatment.

MEDLINE (PubMed) and EMBASE (Elsevier) were systematically searched from inception to September 9, 2025. The following search terms (and variations of these terms as well as ACEi/ARB names) were used: human, mouse, rat, rodent, angiotensin converting enzyme inhibitor, angiotensin receptor blocker, gut microbiome, and gut dysbiosis. The resulting citations were uploaded into Rayyan, a web-based and mobile application screening tool (20). Three authors (ASM, EW, KG) independently screened articles by titles and abstracts using the defined inclusion and exclusion criteria with ASM and EW independently screening and KG serving as a tie-breaker. Full-text articles were then screened by the previously mentioned criteria and desired outcomes. Relevant data were independently extracted using a standardized form and disagreements were resolved through discussion by investigators. A consensus for disagreements (i.e. differing interpretations of outcome data) was ultimately achieved via a third team member serving as a tie-breaker (KG). Quality assessment of animal studies was performed using the SYRCLE (Systematic Review Centre for Laboratory animal Experimentation) risk of bias tool and the human study using the Cochrane Collaboration’s risk of bias tool (21, 22).

## Results

**Figure 1:**
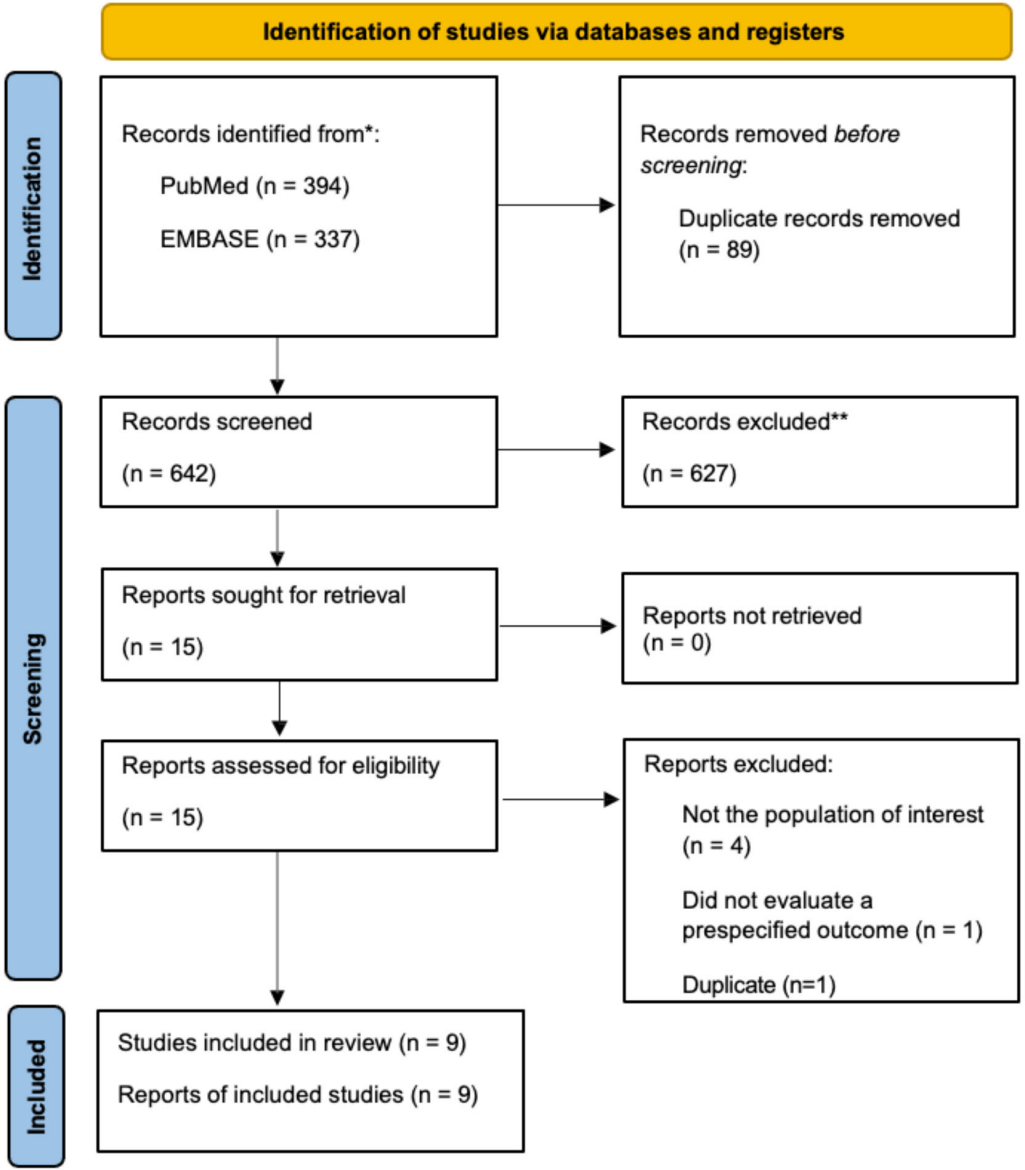
Study selection flow diagram. Flow of information through the different phases of the systematic review according to the preferred reporting items for Systematic Reviews and Meta-Analyses (PRISMA) guidelines.

**Figure 1** summarizes the process of literature selection. A total of 642 articles were initially retrieved after removing duplicates. Fifteen articles remained after title and abstract screening. Four of the twelve articles were excluded because subjects were not the population of interest (i.e. did not have hypertension or were assessing hypertension in the setting of additional comorbidities), one was excluded because it did not evaluate a pre-specified outcome, and one was excluded because it was a duplicate (23–28). After full-text screening, nine articles met the inclusion criteria (17, 18, 29–35). Eight of the nine articles were conducted in rats (17, 18, 29, 31–35), and one study was in humans (30). **Table 1** summarizes the effects of RAS inhibitors on gut composition, function, and associated pathologies in the included studies. A summary of bias risk is shown in **Supplementary Table 1**.

**Table 1:** Effect of RAS inhibitors on gut composition, function, and pathologies in human and animal studies.

Author (year)	Population/model	Intervention/dose (mg/kg/day)/duration (weeks) (administration route)	Change in microbial diversity	Change in B/F ratio	Change in microbial taxa	Change in SCFA (fecal)	Effect on gut permeability	Effect on inflammatory markers (site)
Dong et al. (30)	Human	ACEi or ARB/ NR/NR (oral)	No change	NR	Increased *Odoribacter, Clostridium* and decreased *Staphylococcus, Enterobacter, Klebsiella*	NR	NR	NR
Wu et al. (31)	Rat/DOCA-salt sensitive model	Captopril/50/5 (oral)	No change	No change	Increased *Bifidobacterium, Akkermansia*, and *Blautia*	Increased butyrate and propionate^a^	NR	Reduced IL-6 and TNF-alpha (brain)
Yang et al. (33)	Rat/SHR	Captopril/250/4 (oral)	Increased	NR	Increased *Allobaculum* and *Parabacteroides*	NR	Improved (decrease I-FABP and increase in epithelial health)	NR
Wu et al. (17)	Rat/SHR	Candesartan/1/2-14 (oral)	Increased	Decreased	Increased *Lactobacillus*	Increased acetate, propionate and butyrate^b^	Improved (increased expression of tight junction proteins^c^ and serum LBP)	NR
Robles-Vera et al. (18)	Rat/SHR	Losartan/20/5 (oral)	Increased	Decreased	Increased proportion of strict anaerobes and SCFA producing-bacteria	NR	Improved (increased expression of tight junction protein)	Reduced endotoxins, IL-6, and TNF-alpha (plasma). Reduced Th17/Treg ratio (lymph node)
Dong et al. (29)	Rat/SHR	Losartan/20/12 (oral)	Increased	Decreased	Increased *Alistipes*, *Bacteroides*, *Bifidobacterium*, *Butyricimonas* Decreased *Ruminococcaceae*, *Streptococcus*, *Turicibacter*	NR	NR	NR
Qi et al. (32)	Rat/SHR	Valsartan/7.4/6 (intragastrally)	Decreased	No change	Increased *Lactobacillus*	Decreased isobutyrate and isovalerate	Not improved	Decrease CRP
Xiong et al. (34)	Rat/SHR	Irbesartan/27/8	Increased	Decreased	Increased *Lactobacillus*	Increased acetate, propionate, butyrate, and valerate	Improved (increased expression of tight junction proteins)	Reduced TNF-alpha, IL-6, and IL-1β
Gonzalez-Correa et al. (35)	Rat/SHR	Captopril/85/5 (oral)	No change	No change	Increase strict anaerobes including acetate-producing bacteria and *Parabacteroides*	NR	Improved (increased expression of tight junction protein and decreased I-FABP)	Reduced TNF-alpha, IL-1β, IL-6, TLR4 (brain)

Summary of included studies evaluating the impact of angiotensin-converting enzyme inhibitors (ACEis) or angiotensin receptor blockers (ARBs) on the gut microbiome and related gut pathologies. Diversity changes are reported for alpha and/or beta diversity as defined in the original studies. B/F ratio, Bacteroidetes/Firmicutes ratio; SCFA, short-chain fatty acid; SHR, spontaneously hypertensive rat; DOCA, deoxycorticosterone acetate; NR, not reported; LBP, lipopolysaccharide-binding protein; I-FABP, intestinal fatty acid–binding protein; CRP, C-reactive protein; IL, interleukin; TNF-α, tumor necrosis factor-alpha; Th17/Treg, T helper 17/regulatory T cell ratio; TLR-4, toll like receptor. ^a^Not statistically significant. ^b^Only observed with prolonged treatment (14 weeks). ^c^ Tight junction proteins include claudins, occludin, or zonula.

### Study design of included articles

Seven of the eight hypertensive rat models used spontaneously hypertensive rat (SHR) models (17, 18, 29, 32–35), and one study used deoxycorticosterone acetate (DOCA)-fed rats as the hypertensive arm (31). The control arms included normotensive Wistar Kyoto (WKY) rats or SHAM (defined as normotensive) rats. Three of the eight studies conducted in rats evaluated an ACEi (captopril) (31, 33, 35), and the other four studies used an ARB (candesartan, losartan, valsartan, or irbesartan) as an intervention (17, 18, 29, 32, 34). The study durations ranged from 5 to 14 weeks.

The single human study evaluated the impact of ACEis/ARBs on the human gut microbiome using a single-center, observational study design that included 55 patients with hypertension (30). Hypertensive patients were categorized as (1) untreated, defined as newly diagnosed with hypertension and naïve to antihypertensive therapy (n = 19), or (2) treated, defined as patients with hypertension on ACEi/ARB therapy for at least 4 weeks (n = 36) (30). Patients in the treated group were classified as either well-controlled (n = 24) if systolic blood pressure (SBP) was < 140 mm Hg and diastolic blood pressure (DBP) < 90 mm Hg or as poorly-controlled (n = 12) if SBP was ≥ 140 mm Hg and/or DBP was ≥ 90 mm Hg (30).

### Changes in gut microbial composition and function

In rat models, ACEi or ARB treatment increased gut microbiome diversity (alpha and/or beta diversity) in 62.5% (5/8) of the trials (17, 18, 29, 33, 34). Of the remaining studies, Wu et al. and Gonzalez-Correa et al. reported no significant changes in microbial diversity (31, 35), and Qi et al. observed a decrease in both alpha and beta diversity (32). Notably, Wu et al. utilized a DOCA-salt-induced hypertensive rat model (31), whereas all other animal trials used the spontaneously hypertensive rat (SHR) model (17, 18, 29, 32, 33). Importantly, all studies (8/8) findings suggested that RAS inhibition restored intestinal O_2_ hemostasis (17, 18, 29, 31–35). This was characterized by an increased abundance of strict anaerobes, including SCFA-producing genera such as *Bifidobacterium*, *Bacteroides*, *Blautia*, and *Akkermansia*. Additionally, three trials reported an increase in *Lactobacillus* abundance, a genus known for anti-inflammatory properties (17, 32, 34). Robles-Vera et al. linked gut microbial composition to ARBs’ antihypertensive effect (18). In this study, fecal microbiota transplantation (FMT) from losartan-treated SHR donors to SHR recipients resulted in a significant reduction in BP. In humans, treatment with RAS inhibitors did not significantly impact gut diversity indices (30). However, it increased the abundance of strict anaerobes while decreasing facultative aerobic pathogens, such as *Staphylococcus* and Enterobacterales.

Four animal studies investigated the effects of RAS inhibitors on gut functionality, focusing on change in SCFA production (17, 31, 32, 34). Overall, prolonged RAS inhibition increased fecal levels of linear SCFAs (acetate, propionate, and butyrate), byproducts of fiber fermentation with immune modulatory and vasodilatory effects (17, 31, 34, 36). Conversely, it reduced branched SCFAs (isobutyric and isovaleric acids), byproducts of protein fermentation linked to impaired colon health (32, 37). Collectively, these findings highlight the potential of RAS inhibitors to correct gut microbial composition and functionality. By restoring colonic hypoxia, RAS inhibitors promote SCFA-producing taxa and reduce facultative aerobic pathogens.

### Changes in gut pathologies

RAS inhibition resulted in significant improvements in gut barrier integrity and/or a reduction in inflammatory markers in seven animal studies (17, 18, 31–35). Treatment with ACEi or ARB increased the expression of key tight junction proteins critical for maintaining gut barrier function, including claudins, occludin, and zonula occludens-1, while also promoting an increase in goblet cell numbers and villi length (18, 33). These structural changes collectively contributed to enhanced gut integrity and decrease endotoxemia. Additionally, RAS inhibition restored immune homeostasis by reducing the Th17/Treg ratio and proinflammatory signaling in different organs (18). However, one study by Qi et al. reported that treatment with valsartan failed to restore gut integrity, assessed by changes in zonula occludens-1 expression (32). Notably, plasma levels of Ang II remained persistently elevated during this trial. Finally, hydralazine was shown to significantly reduce BP but had no effect on markers of gut integrity or inflammation (18). These findings underscore the importance of RAS inhibition in preserving gut barrier function and mitigating inflammation, independent of its antihypertensive effects.

## Discussion

The impact of antihypertensive agents on the gut microbiome remains poorly understood. To the best of our knowledge, this is the first systematic review to address this gap. Overall, RAS inhibitors seem to modulate both systemic and local RAS signaling, thereby ameliorating intestinal inflammation and epithelial damage and ultimately improving gut microbial composition and functionality. Ang II, acting through the AT1 receptor, is the most potent effector of the RAS, driving intestinal inflammation, increasing gut permeability, and contributing to gut dysbiosis (13). Elevated colonic levels of Ang I and II have been directly associated with disease severity in Crohn’s colitis (38). Importantly, interventions targeting the RAS, such as ACEi or ARBs, have demonstrated therapeutic benefits that extend beyond BP reduction (39).

**Figure 2:**
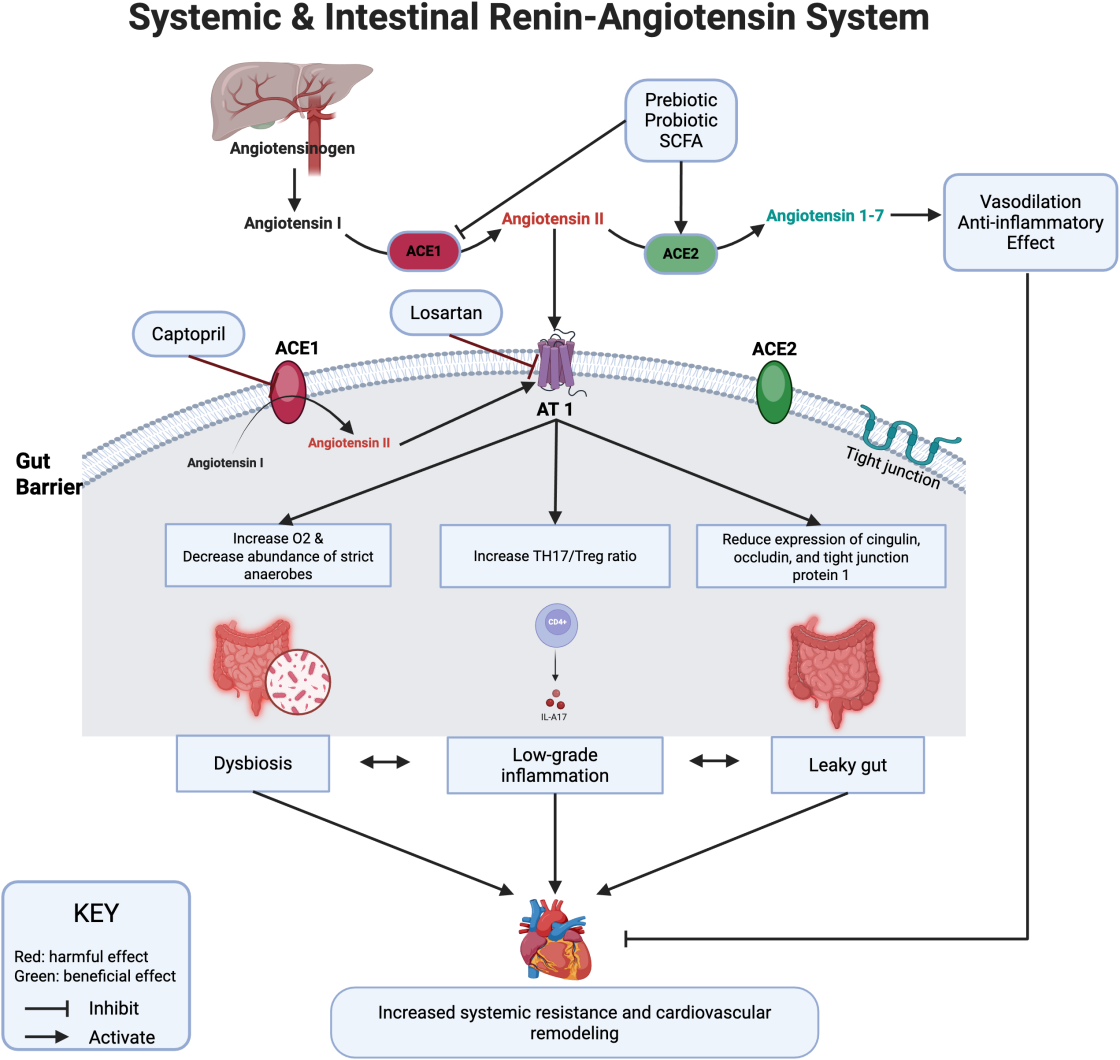
Schematic representation of the bidirectional relationship between Renin-Angiotensin System (RAS) activity, gut health, and blood pressure (BP) regulation. Chronic angiotensin II (Ang II) signaling increases gut permeability, inflammation, and epithelial oxygen tension, promoting dysbiosis enriched in facultative aerobes. RAS inhibition with ACEi/ARB restores epithelial hypoxia and barrier integrity, allowing recolonization by strict anaerobes that ferment fiber into short-chain fatty acids (SCFAs). SCFAs act on G-protein–coupled receptors (direct effect) and inhibit histone deacetylases (indirect effect), leading to vasodilation, immune modulation, and improved BP control. Most mechanistic insights are derived from preclinical models.

Retrospective studies have shown that RAS inhibitors are associated with reduced hospitalizations and lower corticosteroid use in patients with inflammatory bowel disease (IBD) (39). At the molecular level, these benefits are likely attributed to a reduction in proinflammatory cytokines, including IL-1β and TNF-α, modulation of immune responses, such as decreasing the Th17/Treg ratio, and restoration of gut barrier integrity (14, 39, 40). The findings of this review align with these observations, supporting the role of RAS inhibitors in improving gut barrier integrity and reducing inflammatory markers. The interplay between RAS, gut pathology and BP regulation is illustrated in **Figure 2**.

Chronic Ang II exposure disrupts gut oxygen and microbial dynamics, leading to a persistent dysbiotic state (14, 41). This effect is likely indirect, driven by a cascade of inflammation and epithelial damage that together increase oxygen availability. Dysbiosis, in turn, perpetuates inflammation and gut barrier dysfunction, creating a cycle that sustains a pro-inflammatory state (**Figure 2**). RAS inhibition interrupts this cycle and re-establishes oxygen homeostasis in the gut, which favors the growth of strict anaerobes such as *Bifidobacterium* and *Akkermansia*. These bacteria are key for SCFA production and mucin recycling, which are essential for immune regulation and gut health (42, 43). In this review, treatment with ACEi or ARBi consistently increased the abundance of these beneficial taxa (18, 31). Interestingly, the interplay between gut dysbiosis and RAS signaling appear to be bidirectional. Butyrate metabolism by colonocytes consume oxygen, leading to a hypoxic epithelial surface (44). Disruption of this hypoxic environment has pathophysiological consequences. In the SHR model, butyrate supplementation mitigated Ang II-mediated hypertension, gut dysbiosis, and barrier dysfunction (14). Prebiotics and probiotics have modulated RAS signaling, suppressing the classical ACE1-Ang II pathway while enhancing the ACE2-Ang 1–7 axis, improving gut function and BP control (45). Similarly, ACEi and ARBs increase Ang-(1–7) signaling through distinct mechanisms. ACEis increase Ang I substrate availability and reduce Ang-(1–7) degradation, whereas ARBs upregulate ACE2 expression, shifting RAS balance toward Ang-(1–7) (46). These mechanisms help explain, at least in part, the antihypertensive and anti-inflammatory effects of pharmacological RAS inhibition and potential synergy with prebiotics.

Key considerations regarding the findings of this review merit attention. First, the interaction between RAS inhibition and the gut microbiome may depend on the pharmacokinetics of specific RASs’ inhibitors (e.g., ACEis/ARBs) and components (e.g., Ang II). While most RAS inhibitors consistently benefited the gut, valsartan showed conflicting results, reducing microbial diversity without improving barrier integrity (32). One hypothesis for this could be attributed to difference in methodology or inherent valsartan properties and would require validation in future studies. Notably, valsartan has lower bioavailability compared to other ARBs, and it was administered intragastrically in this study, unlike the oral administration used in the remaining studies (47). Further research is needed to determine whether the gut benefit of RAS inhibitors is a class effect. Second, the beneficial effects of RAS inhibition appear to involve both systemic and local RAS signaling. Ang II has a longer tissue half-life (~15 minutes) than its plasma half-life (~1 minute), underscoring the significance of local RAS activity (13, 48). Moreover, the most pronounced gut benefits were observed with prolonged candesartan treatment, emphasizing the chronic nature of Ang II-driven gut dysbiosis and pathology (17). It is important to note that Ang II is not inherently harmful. Physiological levels and acute elevations are critical for maintaining vascular tone and fluid balance (49). Third, although the hypotensive effect of RAS inhibitors could improve gut health, it is likely not sufficient. Robles et al. tested this theory, where the administration of hydralazine to SHR rescued BP but did not affect gut composition and colonic integrity, suggesting that the anti-inflammatory effect of ACEi and ARBi is likely RAS dependent. Finally, the influence of Ang II on the microbiome may be affected by experimental factors, such as diet, sampling size, and housing facility (50).

Previous reports have illustrated a complex relationship between antihypertensive medications and the gut microbiota. Our review describes the impact of ACEis/ARBs on gut bacteria, the gut integrity, and inflammation but it should be noted that intestinal bacteria likely play a role on ACEi/ARB biotransformation/pharmacokinetics which may subsequently impact BP effects. This has been previously demonstrated with calcium channel blockers in rats and with human flora (51). How gut bacteria composition impacts ACEi/ARB metabolism has not been fully elucidated, but this missing link should be considered when interpreting the results of this systematic review.

### Limitations

Limitations of this systematic review include a small article sample size and heterogeneity in methodology between studies. Only one human study was identified and carried a moderate risk of bias, which is important to consider with regards to study generalizability. Assessment of bias was in animal studies was limited by missing details on housing and study design. These limitations further highlight the scarcity of available evidence and should be considered in the interpretation of our review. Additionally, non-English language studies were excluded.

## Conclusions

In conclusion, this systematic review highlights the intricate relationship between Ang II, inflammation, gut barrier dysfunction, and microbial dysbiosis, identifying chronic Ang II exposure as a key driver of gut pathology. The findings emphasize the interconnected roles of RAS signaling and gut health, suggesting that targeting both systems may yield synergistic therapeutic benefits. While preclinical studies have shown promise, evidence in humans remains limited. Given the safety and accessibility of dietary interventions like prebiotics, probiotics, and synbiotics, future research should investigate their potential to modulate RAS signaling, restore gut homeostasis, and improve blood pressure control.

## Data Availability

The original contributions presented in the study are included in the article/supplementary material. Further inquiries can be directed to the corresponding author.
