# Supplementary material for: Angiotensin converting enzyme inhibitors and angiotensin receptor blockers impact on the gut microbiome: a systematic review
Source: Front Endocrinol (Lausanne). 2025 Oct 23;16:1685424. doi: 10.3389/fendo.2025.1685424 (PMC12588817; doi:10.3389/fendo.2025.1685424)
Supplement: Supplementary file 1 [file Table1.docx]

Risk of Bias: Animal Studies

| **Study** | Sequence Generation | Baseline Characteristics | Allocation Concealment | Random Housing | Blinding (Intervention) | Random Outcome Assessment | Blinding (Outcome) | Incomplete Outcome Data | Selective Outcome Reporting | Other Sources of Bias |
| --- | --- | --- | --- | --- | --- | --- | --- | --- | --- | --- |
| Wu et al. (2022)^30^ | ? | + | ? | ? | ? | ? | ? | N.A. | + | + |
| Yang et al.  (2019)^32^ | ? | + | ? | ? | ? | ? | ? | N.A. | + | + |
| Wu et al.  (2019)^17^ | ? | + | ? | ? | ? | ? | ? | N.A. | + | + |
| Robles-Vera et al.  (2019)^18^ | ? | + | ? | ? | Y | ? | Y | N.A. | + | + |
| Dong et al.  (2023)^28^ | ? | + | ? | ? | ? | ? | ? | N.A. | + | + |
| Qi et al.  (2021)^31^ | ? | + | ? | ? | ? | ? | ? | N.A. | + | + |
| Xiong et al. (2024)^34^ | ? | + | ? | ? | ? | ? | ? | N.A. | + | + |
| Gonzalez-Correa et al. (2024)^35^ | ? | + | ? | ? | Y | ? | Y | N.A. | + | + |
| Abbreviation: (+) indicates low risk of bias; (-) indicates high risk of bias; (?) indicates unclear risk of bias; N.A. = Not applicable | | | | | | | | | | |

Risk of Bias: Human Study

| Study | Bias due to confounding | Bias in selection of participants into the study | Bias in classification of interventions | Bias due to deviations from intended interventions | Bias due to missing data | Bias in measurement of outcomes | Bias in selection of the reported results | Overall Bias |
| --- | --- | --- | --- | --- | --- | --- | --- | --- |
| Dong, 2022 | Moderate | Low | Low | Low | Low | Low | Low | Moderate |
